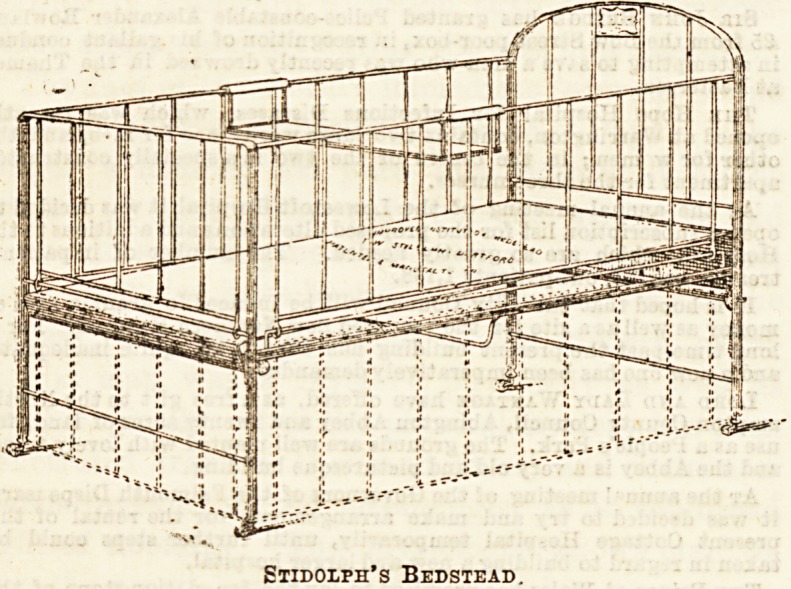# Children's Cots

**Published:** 1892-11-05

**Authors:** 


### PRACTICAL DEPARTMENTS.

### II.—CHILDREN'S COTS (*continued*).

The collapsible cot has many good points to recommend it for institution use. It can be put away or got ready in a very few moments, and it takes but little room when not required. The simplicity of the contrivance is greatly in its

ATKINSON'S COLLAPSIBLE COT (OPEN).

favour, and we think that Messrs. Atkinson are to be congratulated on their invention.

The bedding used for children should receive as much

ATKINSON'S COLLAPSIBLE COT (CLOSED).

consideration as the cot itself, and is of even more importance. Restless and unrefreshing sleep is often produced by improper mattresses and coverings. Of course, children sleep, as people are fond of saying, "anyhow and anywhere," and so they do, simply because a stage is reached when they cannot keep awake; but their slumbers would be more beneficial if their surroundings were more wisely considered. All beds composed of feathers are inadmissible in this hygenic age, and when funds permit it, good horse-hair mattresses are not only the best, but also the cheapest in the end. They can be done up and put into fresh ticks, and made equal to new as often as may be required of them. Certain other things are cheaper to buy, and they have to be used where horsehair cannot be afforded. Then, again, as regards the spring bottoms, of course the old rails may be preferred, and can be had to any of the cots we mention, but they are in many respects less desirable than these delightful springs. In the case of patients with fractured limbs, when a firm resting place is essential, a light, even board can be slipped under the mattress to give the requisite support, or a really good straw mattress may be preferred. We have spoken of the advantages and disadvantages of movable sides, but the ends of the cots should also be considered, and when we leave convalescent or healthy children safely deposited in cots whose shape is of less consequence than their size, we may bestow a little special attention on the accommodation for surgical and accident cases. Here the sides must always be adjustable, and the method of attaining this end should be as simple as possible—no jarring should be possible. To see the side of a cot needing shaking or jerking into place whilst

Stidolph's Bedstead.

a sufferer from hip disease lies on the mattress is to witness a terrible addition to the pain which is the inevitable portion of the owner of that diseased limb. Again, in cases of fractures, such movements are even more disastrous, although not more uncomfortable. Therefore, the motion by which the sides are raised or lowered should be perfectly smooth and absolutely silent. Doctors often administer anæsthetics to children lying in their beds, and this is a great gain to the more sensitive ones, who thus escape the nervous anticipations attending transfer elsewhere, and it is essential, or at any rate desirable, for the headpiece of the cot to be removed. It ought to come away so quietly that no sound is heard, and the doctor is then conveniently situated for administering his chloroform or ether. The footpiece has often to be got rid of, so as to leave the surgeon freedom for setting fractures or for any operation which can best be performed by him from a position at the foot of the cot, and therefore this must be allowed for in furnishing a ward for accidents.

Messrs. Stidolph, of Dartford, have a useful iron bedstead, which we give here. Double navy sacking forms the bottom of the cot, and it is strained into absolute firmness by a very ingenious arrangement, which we propose to show when considering full-sized beds for institution use. It is certainly a good idea to have a material which can be washed, and thus purified promptly whenever occasion arises; and, of course, extra supplies of the cloth-sacking would be always kept in readiness in an institution.

(*To be continued.*)

## Figures and Tables

**Figure f1:**
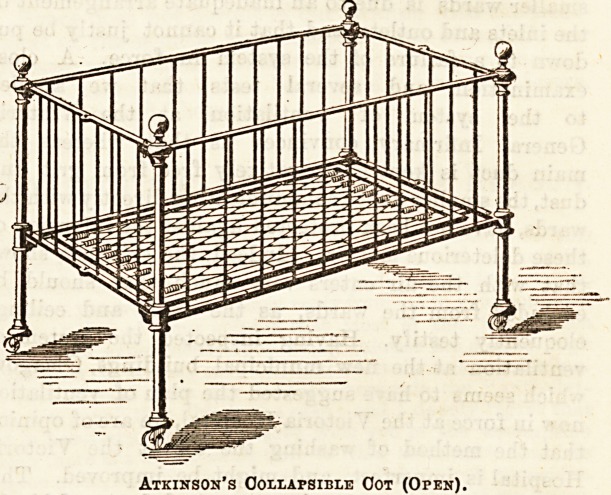


**Figure f2:**
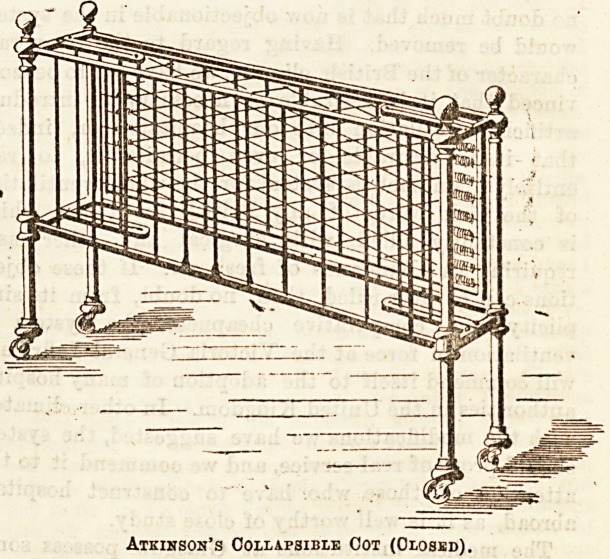


**Figure f3:**